# Beyond Cortical Signatures: Measurement Gaps Across Theories of Consciousness

**DOI:** 10.3390/brainsci16101087

**Published:** 2026-10-09

**Authors:** Maria Svet, Arosh S. Perera Molligoda Arachchige

**Affiliations:** 1 Independent Researcher, 1205 Geneva, Switzerland; 2 University of Milan, Via Festa del Perdono 7, 20122 Milan, Italy

**Keywords:** consciousness, adversarial collaboration, conscious access, action-policy competition, interoception, valence, metacognition, cognitive neuroscience

## Abstract

Adversarial testing has strengthened the empirical standards of consciousness science, but recent results also expose a measurement problem: competing theories frequently make predictions about the same neural quantities and can therefore be challenged without being decisively discriminated. This evaluative review incorporates a purposive structured audit of contemporary theories and theory-relevant experiments rather than a systematic census. The audit separates trial-level from origin-level frameworks, explicit from interpreted commitments, and positive from stated-null predictions. Policy count and incompatibility remain sparsely occupied coordinates, whereas organism-wide cortical-autonomic coherence is not explicitly formulated as an access criterion by the trial-level accounts and is therefore treated as a sensitivity-dependent gap rather than an unqualified absence. Importantly, an unoccupied coordinate is not itself a discriminating prediction: direct theoretical adjudication requires opposed commitments. In the baseline theory-addressed content-bearing sample, valenced content is absent; under the most permissive sensitivity rule, it rises to 6 of 57 experiment rows (4 of 30 publications). The crossed conflict-by-valence experiment specified here can be run now, but it does not discriminate among theories that are silent on those coordinates; the autonomic-only condition that would generate a genuinely opposed test cannot yet be run because a validated report-independent measure of interoceptive access is lacking.

## 1. Introduction

For thirty years, the standard complaint about consciousness science was that its theories were not tested. The complaint has been answered, and the answer was worse. The Cogitate consortium ran a preregistered adversarial test of global neuronal workspace theory (GNWT) and integrated information theory (IIT) across fMRI, MEG and intracranial EEG in 256 participants, with pass and fail criteria agreed in advance by proponents of both theories and by a theory-neutral consortium [1], and reported that neither central prediction survived intact [2]. GNWT was challenged by the absence of prefrontal ignition at stimulus offset—a null that cannot be blamed on insensitivity, since offset responses were robust elsewhere and the same prefrontal sites showed strong onset responses to the same stimuli. IIT was challenged by the absence of sustained within-posterior synchronisation. The consortium declined a verdict, adopting a Lakatosian soft-falsification frame, and GNWT proponents have published their own reading of the same data [3].

The usual lesson drawn is about the theories: too vague, too unfalsifiable, too well defended by auxiliary assumptions. The more consequential lesson is about the measurements, and the consortium points at it: the prefrontal prediction is shared by those higher-order theories that treat the prefrontal cortex as a supplier of visual content rather than merely as what makes content conscious, and the posterior prediction is shared by recurrent processing theory. An adversarial test, on this reading, does not punish a theory; it punishes a class of prediction. A theory whose distinctive prediction happens to be the one everyone else also makes cannot be distinguished by the experiment that tests it, however well that experiment is run.

That is the thesis of this review. The problem is not simply that theories of consciousness are untested; it is that many are tested on the same quantities. Two distinct structures must therefore be separated. A discriminating coordinate is one on which at least two theories make opposed predictions; an unoccupied coordinate is one that the sampled theories leave unspecified. The former can adjudicate between theories, whereas the latter identifies underexplored measurement space but cannot, by itself, discriminate among theories that remain silent.

Locating these structures requires knowing what theories actually commit to, rather than what review-article summaries attribute to them. We therefore conducted a purposive structured audit rather than a census or systematic review. The revised coding separates trial-level accounts from origin-level frameworks, separates previously compound rows where their commitments diverge, and codes ten measurable domains using source-anchored categories of EXPLICIT, INTERPRETED, NOT_LOCATED, NOT_APPLICABLE, and UNRESOLVED, with polarity recorded separately. The complete cell-level justifications and study inventory are provided in the Supplementary Materials.

Three conventions govern what follows. This is an evaluative review, not a census: claims from the structured audit apply to the purposively sampled accounts and studies and are not prevalence estimates for the field as a whole. We distinguish claims about access from claims about phenomenal experience, and we distinguish absence of a located commitment from an explicit null prediction. Organism-wide cortical-autonomic coherence (M5) is treated as a candidate measurement coordinate rather than built into the definition of access; related brain–body measurements are reviewed explicitly below, and the coding question is limited to whether a theory formulates joint cortical–autonomic coherence as an access criterion.

The review proceeds from conceptual bookkeeping to measurement. Section 2 asks which distinctions the field has earned. Section 3, Section 4 and Section 5 evaluate biological self-maintenance, action arbitration, and valence as organism-wide reconfiguration. Section 6 maps disputes between theories and what the adversarial test removed from the table; Section 7 presents the coding results; Section 8 examines three candidate dissociations; Section 9 states the minimal measurement programme. A separate companion paper develops an author-derived theoretical framework related to some of these coordinates; that framework is not used as evidence for the conclusions of this review.

## 2. Earned and Unearned Distinctions

Any claim about consciousness is a claim about one of at least seven things, and most disputes here are about which one. The coding in Section 7 depends on holding them apart: unconscious processing; conscious content; access; online metarepresentation of one’s own processing; control; post hoc reconstruction; the putative bearer of phenomenal states. These are not degrees of one variable but dissociate pairwise.

The oldest distinction is also the least stable. Block’s separation of phenomenal from access consciousness [4] made the inference from “globally available” to “felt” a step requiring argument, later given empirical teeth by the overflow claim that experience contains more than the system can report [5,6]. The programme that grew from it—isolating the correlates of conscious content from its prerequisites and consequences [7], and distinguishing conscious state from content [8]—is a genuine achievement, and the no-report literature of Section 6 its most valuable product.

What has changed is confidence in the two-kinds framing. An argument from inside the experimental camp holds that dividing consciousness into two kinds converts a graded multi-component phenomenon into two entities, one inaccessible to measurement by construction [9]. That is a conceptual paper, not a result, but it converges with an independent movement toward feature profiles: multidimensional assessment of animal sentience replacing a single scale [10], indicator lists for invertebrates [11], and a framework treating consciousness as a profile of separately assessable capacities [12].

The field has earned the process/content/access distinctions and has not earned the phenomenal/access dichotomy in its strong form, and the two are usually run together to the detriment of both. The first set is earned because each distinction is independently measurable and has produced dissociations that survived attempts to explain them away: integration without awareness [13], frontal correlates tracking report rather than the percept [14], and conscious state varying independently of content [8]. The dichotomy is not, because its central claim—a second kind of consciousness whose presence comes apart from access—is what no measurement can establish; overflow arguments turn on the interpretation of partial report, the opposing position denies the separation is coherent [15], and an intermediate view treats access as partial and hierarchical rather than all-or-none [16].

The cost of conflating these levels shows up in how theories get evaluated. A theory specifying when content becomes globally available answers an access question, and its success says nothing directly about phenomenality; but with the dichotomy in the background, such theories are read as accounts of phenomenal experience, criticised for failing a task they never took on. The converse error is as common: theories staking a claim on phenomenality are credited with the access evidence. Feature profiles help by forcing each claim to name its own observable, which is what makes the coding of Section 7 possible.

## 3. From Regulation to a Minimal Bearer

The literature on biological self-maintenance explains how an organism maintains its own boundary and regulates viability, but generally stops short of deriving experience. Autopoiesis treats cognition as structural coupling through self-producing organisation [17]; Di Paolo’s account of adaptivity adds regulation relative to viability boundaries [18], and later work formalises agency through individuality, normativity, interactional asymmetry, and active inference [19,20]. Basal-cognition approaches similarly catalogue sensing, valuation, memory, learning, decision, and anticipation without requiring phenomenal experience [21,22]. A parallel evolutionary line frames early nervous systems as devices for whole-body coordination, with minimal cognition understood as sensorimotor coordination [23,24]. These programmes therefore supply a rich account of organisation and control while leaving the transition to felt experience open.

Proposals diverge over where experience begins. Cellular and bioelectric accounts retain a functional vocabulary of distributed problem-solving, with the effective ‘self’ defined by the scale over which information and control are coordinated [25,26], whereas radical cellular accounts extend sentience to single cells [27]. Arguments about plant consciousness illustrate the methodological difficulty: some objections appeal to absent neural structures or to failures of the empirical evidence offered for plant cognition [28,29], while replies challenge the assumption that nervous tissue is a prerequisite [30,31]. The durable lesson is methodological rather than taxonomic: behavioural flexibility alone cannot identify the architecture that produces it, because similar behaviours can be generated by systems of very different internal complexity [32].

More conservative evolutionary proposals place the threshold at specific integrative capacities. Feinberg and Mallatt emphasise isomorphic neural representations and hierarchical sensory maps [33]. Ginsburg and Jablonka propose unlimited associative learning as a marker of the transition, developing it into explicit empirical predictions and a vertebrate-workspace account [34,35,36,37]. Homeostatic accounts instead relate feeling to regulation of the internal milieu and affective control [38,39,40,41]. These approaches disagree about the relevant threshold, but all move beyond bare self-maintenance by requiring some form of organised integration rather than merely the persistence of a boundary.

Anti-corticocentric proposals push the same point further. Merker locates a candidate core of the conscious state in upper brainstem and mesodiencephalic systems that integrate body schema, action space, and motivational ranking, with cortex supplying much of the content [42]; analogous arguments have been made for insects [43]. The New York Declaration on Animal Consciousness and subsequent discussion of a shifted null hypothesis reflect the resulting willingness to treat non-cortical architectures as serious candidates [44,45]. These positions matter here because they warn against making any one human cortical signature a definition of access.

Across these programmes, single-body integration and control recur, and transitions in cognitive evolution have also been described as changes in control regime [46]. However, the literature does not establish that the number or heterogeneity of conflicts determines conscious access. That stronger proposition belongs to the authors’ separate companion framework and is not treated here as a result of the review. For the present audit, policy count and incompatibility are therefore candidate measurement coordinates whose evidential status depends only on what the coded theories themselves state. The review uses these studies to motivate measurement, not to infer a new theory from their convergence.

## 4. Arbitration as a Function

Action arbitration supplies a useful stress test because competition over one body can be solved without conscious access. The basal ganglia implement channel selection through disinhibition and surround suppression, allowing one action channel to be released while competitors are inhibited [47,48,49,50]. Evidence-accumulation models reproduce choice accuracy and reaction-time distributions by integrating evidence to a threshold, with urgency signals adjusting when commitment occurs [51,52,53,54]. Affordance-competition models likewise maintain several possible actions simultaneously in fronto-parietal circuits until value and context bias one toward execution [55,56,57,58,59,60]. These mechanisms show that competition itself is not sufficient for conscious access.

Supramodular interaction theory (SIT) makes a narrower claim. It proposes that phenomenal states permit interaction among otherwise encapsulated response systems when they compete for the same skeletomotor output [61,62,63]. Passive frame theory further separates conscious contents from the processes that select among them [64]. On this view, the relevant distinction is not simply ‘conflict versus no conflict’ but the location and effector class of the conflict: skeletal action is treated differently from processes resolved wholly outside the somatic output system.

The empirical support is primarily taxonomic and behavioural. Conflicts such as pain-for-gain, breath holding, or delay of gratification are contrasted with conflicts that are said to remain introspectively impenetrable. In laboratory work, participants sustaining incompatible skeletomotor intentions report stronger subjective effects than under compatible conditions, while smooth-muscle controls do not show the same pattern [65,66,67]. Neuroimaging support is more limited, with reported associations including the somatosensory cortex rather than a clear executive signature [67]. The smooth-muscle comparison is especially important because without it the evidence would support only a generic conflict account.

The theories are also bounded by their authors. SIT concerns integrative processes that purportedly require phenomenal mediation, not every process whose output can become conscious [62]. Critiques question skeletal-muscle specificity, the degree of encapsulation, the treatment of affect, and whether voluntary control evolved primarily for external action or also for regulation of internal-state conflicts [68,69,70,71,72]. Passive-frame arguments, meanwhile, stress that conscious contents can be present without themselves performing selection [73,74,75,76]. These disagreements make the effector boundary more informative than policy count alone.

The resulting inference is deliberately limited. Existing unconscious arbitration mechanisms show that policy competition alone does not entail access; they do not establish that conflict is unnecessary. SIT and passive frame theory are unusual because they state or imply commitments over where conscious conflict should and should not occur. In the audit, those commitments are kept distinct from silence by other theories. The genuinely adversarial cell is therefore the autonomic-conflict boundary, not the mere manipulation of policy count or incompatibility, and even that cell requires caution because the positive opposing commitments are interpreted rather than explicit.

## 5. Valence and Organism-Wide Reconfiguration

Valence provides a second bridge between regulation and action. Long before contemporary consciousness theories, motivational accounts proposed a common currency through which otherwise incommensurable goals could be compared [77,78]. Interoceptive work subsequently reframed the body from a monitored object to a control loop: visceral signals contribute to perception, memory, decision, emotion, and behavioural regulation [79,80,81,82,83,84]. Allostatic accounts add anticipatory control, in which expected needs alter regulatory targets before a deviation occurs, and large-scale visceromotor networks provide an anatomical substrate for this body-brain coordination [85,86,87].

Brain-body coupling is also evident at shorter timescales. Respiratory phase can modulate perceptual sensitivity: in a 2026 near-threshold visual-detection study, sensitivity was higher during inspiration, alongside respiration-linked changes in cortical excitability, arousal, and directed information flow [88]. This finding is adjacent to the heartbeat-evoked and visceral-monitoring literature already discussed in Section 7. It is not equivalent to the M5 construct used in our audit, but it demonstrates why cortical–autonomic relations cannot be described as unmeasured or irrelevant to perception.

Emotion theories already describe organism-wide reconfiguration in several vocabularies. Component-process theory emphasises synchronisation of organismic subsystems [89]; evolutionary accounts treat emotions as situation-specific operating modes that tune many parameters at once [90,91]; action-readiness traditions stress control precedence [92]; and constructionist accounts recast the same coordination in allostatic terms [93,94]. The convergence is functional rather than mechanistic: these traditions agree that affective states reorganise multiple systems, but not on a single neural implementation.

The empirical signatures are correspondingly heterogeneous. Meta-analyses do not yield stable category-specific neural or autonomic patterns for discrete emotions [95,96], and facial expressions alone poorly identify emotion categories [97]. Formal accounts deriving valence from prediction-error dynamics or deep active-inference hierarchies remain proposals rather than settled results [98,99,100]. Valence is also not unitary: hedonic ‘liking’ and motivational ‘wanting’ can dissociate neurochemically and anatomically [101]. A single positive-negative scalar therefore risks collapsing distinct components that may have different relations to access.

A second constraint is the distinction between integrated regulation and experienced valence. Defensive or appetitive control can occur without establishing that the state is consciously felt [102,103,104,105,106,107]. Pain research provides a useful analogue: nociception, motivational trade-offs, and felt evaluation are separable, and comparative, lesion, and circuit evidence does not support one simple interoceptive locus [108,109,110,111,112,113,114,115,116]. We therefore treat valence as a measurable coordinate without adopting the stronger author-derived claim that conflict heterogeneity determines the dimensionality of valent integration. That proposal belongs to the companion framework; the present review asks only whether coded theories and experiments already make commitments over valence and brain–body integration.

## 6. Theories and Their Empirical Tests

The theories are easier to describe as a list of disagreements than as schools. Almost everyone agrees that conscious content is connected to broad availability; the disagreements are about what makes it conscious—network topology, causal structure, second-order representation, a predictive model of the body. We organise the map by dispute, where the empirical blows of the last five years landed, most striking several theories at once.

### 6.1. Where Content Lives

The localisation dispute—posterior versus prefrontal—was the most productive, because both sides stated mutually exclusive predictions. The workspace line ties access to late global ignition in a network privileging prefrontal cortex [117,118], its strongest evidence of nonlinearity: a second attentional-blink target’s fate is bimodal rather than graded [119], and the masking threshold coincides with a sharp inflection in late components [120]. Notably, GNWT’s rationale for broadcast is flexibility rather than arbitration—an answer to the frame problem [121], a serial router for composite mental programmes [122]. IIT proceeds from phenomenological axioms to maximally irreducible integrated information, placing the hot zone in posterior temporo–parieto–occipital cortex [123,124], experience identified with the distinctions and relations unfolded from a substrate [125]. Recurrent processing theory holds instead that local recurrent activity in sensory cortex makes content conscious, entailing phenomenal content without access [126,127]. The dispute was long reproduced positionally [128], with the well-founded suspicion that outcomes depend on method and on who runs the experiment [129].

### 6.2. Who Predicts Phenomenality

Few theories predict phenomenality rather than access, and the price is metaphysical load or denial of the explanandum [130,131]. Higher-order theories predict structurally: being conscious is constituted by a second-order representation, entailing dissociations of metacognitive sensitivity with first-order processing intact [105,106]; the most operationalised variant nests a one-dimensional awareness state above a high-dimensional perceptual space [132], with consequences for confusing perception and imagination [133]. Attention schema theory is more radical: a system attributes awareness to itself because it models its own attention [134,135], with an attempt to reconcile AST, GNW, HOT, and illusionism [136]. Predictive processing explains not the presence of experience but its structure—perception as controlled hypothesis [137], interoceptive inference as the basis of emotion and bodily selfhood [82,138]—claiming only a basis for correlate-hunting [139]. The poles bound the space: the hard problem as non-derivability of experience from function [140], the meta-problem with illusionism as its answer [141]; the working stake is more modest, making the hard problem operationally easier through accumulated structural correspondences [142].

The methodological threat is logical rather than empirical. The unfolding argument shows that if consciousness is defined by causal structure, a functionally equivalent unfolded network without recurrence behaves identically, including in all reports about experience, while receiving a different value of the measure—so the theory is either false or indistinguishable from its opposite by any behavioural experiment [143]. Responses are divided between rejecting its behaviourism [144] and noting a hidden premise about brain states [145]. The problem is deeper than any one theory: falsification requires independent access to phenomenality, and without that bridging assumption every theory is protected from refutation [146]—hence criteria for empirical theories [147], a Lakatosian accounting scheme [148], separating a testable weak core from a loaded strong one [149]. The 2023–2025 controversy over whether IIT is pseudoscientific continues the same dispute, and we report it without taking a side [150,151].

### 6.3. Access Without Report

Dependence on report biases toward frontal theories, and the key result is direct: in binocular rivalry, frontal activity tracks introspection and the act of reporting rather than the percept [14]. The programmatic statement [152] and the isolation of perceptual from report-related correlates [153] extend to a report-independent bifurcation signature [154], large-scale networks in a no-report visual paradigm [155] and audition [156]. Calibration is needed both ways: report reliably adds frontal correlates, but null frontal results do not themselves bury prefrontal theories [157], and no-report designs have their own defect—without report one does not know what the participant experienced [158].

### 6.4. What Cogitate Removed

Exactness matters, because the loose version circulates widely. For IIT, the most direct challenge was the absence of sustained within-posterior synchronisation; its content-maintenance prediction passed the pre-set criteria—sustained responses reflecting stimulus duration appeared in occipital and lateral temporal cortex—but sustained representation of orientation did not, although orientation decoded reliably in all three modalities. For GNWT, the principal failure was the absence of ignition at stimulus offset, with a secondary challenge on content: category decoded in prefrontal cortex independently of task, identity not at all, orientation only in MEG—raising the question whether prefrontal cortex broadcasts all conscious content or only an abstract part [2].

The adversarial test succeeded methodologically and failed theoretically, and that combination is the most important fact currently available about the field. It succeeded where it matters: predictions fixed in advance, criteria agreed by opponents, results reported against them rather than reinterpreted afterwards. It failed in that the outcome was not fewer live theories but less confidence in a shared dependent variable. The structural lesson is the one the consortium states and the field has not absorbed: a prediction shared with competitors is no asset, because the experiment testing it tests everyone at once. What a new theory can most usefully offer is a prediction about a quantity current designs hold constant—and whether such a quantity exists is answerable by coding the theories.

## 7. The Measurement Gap

### 7.1. What the Field Currently Measures

Theories of consciousness are compared predominantly on where and when conscious contents are encoded. Section 6 traced the consequence: an adversarial test can challenge a shared prediction without uniquely adjudicating among theories that share it. To examine the breadth of this problem, we used a purposive structured audit of 12 theory accounts across 10 measurable domains, with trial-level and origin-level frameworks analysed separately, and a publication/experiment inventory of theory-relevant empirical work (Table 1 and Table 2; full cell-level data in Tables S1–S4).

### 7.2. Coding Procedure

The account set was selected purposively rather than as an exhaustive census of consciousness theories. It was designed to span influential contemporary accounts with sufficiently explicit empirical commitments for source-anchored coding. Trial-level theories of conscious access were distinguished from origin-level frameworks concerned principally with the phylogenetic emergence or distribution of consciousness. Composite rows in the submitted version were separated where their commitments diverged (HOT from HOSS; SIT from passive frame theory). The ten domains were M1, number of simultaneously competing action policies; M2, incompatibility of competing policies; M3, valence; M4a, access to interoceptive content as content; M4b, visceral modulation of access to exteroceptive content; M4c, conscious involvement in purely autonomic conflict; M5, organism-wide cortical-autonomic coherence; M6, dimensionality of phenomenal structure; M7, metacognitive sensitivity; and M8, cortical signature of access.

Each cell was coded EXPLICIT when a source states a prediction over the domain, INTERPRETED when the commitment follows from the source with limited interpretation, NOT_LOCATED when no such commitment was found in the searched primary material, NOT_APPLICABLE where the domain does not apply to the account, or UNRESOLVED where evidence was insufficient. Polarity was coded separately as positive, stated-null, or negative. Three coordinates in the submitted scheme—M1 (policy count), M2 (policy incompatibility), and M5 (organism-wide cortical-autonomic coherence)—overlap with variables developed by the authors in a separate companion framework; this is disclosed because their inclusion is not theory-neutral. Table S3 therefore repeats occupancy coding against the independently developed ConTraSt dimensional scheme. All 12 ConTraSt dimensions were occupied by at least one sampled trial-level theory, with explicit YES classifications ranging from 1 to 7 of 10 accounts; the analysis therefore shows that apparent sparsity depends on the coordinate system and does not independently validate M1, M2, or M5.

A second human coder (the second author) independently recoded the complete 120-cell theory-by-domain dataset using the frozen codebook and cited primary-source loci before consensus resolution. Although familiar with the manuscript as a co-author, the second coder did not view the first coder’s completed cell-level coding table or the companion framework before completing the independent recoding. Pre-adjudication raw agreement was 115/120 cells (95.8%). Cohen’s κ, computed on the five-category code set (EXPLICIT, INTERPRETED, NOT_LOCATED, NOT_APPLICABLE, UNRESOLVED), was 0.930. The five disagreements were resolved by discussion, with the consensus classifications reported in Table S1. At the study-inventory level, the second coder independently reviewed the 23 baseline publication classifications and the 45 experiment-row classifications used to construct Table 2; pre-adjudication agreement was 23/23 and 45/45, respectively (100% at both levels).

### 7.3. Theories by Measurable Quantity

The revised trial-level audit shows a strong concentration of explicit commitments on M8: six trial-level accounts make explicit positive predictions over the cortical signature of access, with three additional interpreted positive commitments. By contrast, M1 and M2 each contain one explicit positive commitment (SIT), one interpreted stated-null commitment (passive frame theory), and no located commitment in the remaining trial-level accounts. These are therefore sparsely occupied coordinates, not automatically discriminating ones.

The autonomic/interoceptive domain is no longer treated as a single undifferentiated column. M4a, M4b, and M4c separate access to interoceptive content, visceral modulation of exteroceptive access, and conscious involvement in purely autonomic conflict. This separation matters because a stated-null commitment in the supramodular/passive-frame family is conceptually different from silence by other theories, and because predictive-processing and brain–body accounts provide positive commitments or interpretations on adjacent subdomains. Accordingly, the revised analysis reserves the term discriminating for opposed predictions rather than for empty cells.

M5 is also stated more cautiously. No trial-level account in the revised coding gives an explicit prediction over organism-wide cortical-autonomic coherence as defined here, although predictive-processing and neural-subjective-frame accounts receive interpreted positive classifications. The stronger statement that cortical–autonomic relations are unmeasured is incorrect: heartbeat-evoked responses can predict subsequent visual detection, and the neural subjective frame proposal explicitly links visceral monitoring to conscious perception [159,160]. Our narrower claim is therefore only that the specific joint coherence measure used for M5 is not explicitly formulated as an access criterion by the coded trial-level theories.

### 7.4. Content Inventory

The revised inventory distinguishes publications from experiment-level rows and defines theory-addressed status operationally. The earlier figure of 36 ‘studies’ was a legacy working-record count and is not directly comparable with the revised denominator. Four paradigm/theory-source records from that legacy inventory were removed from the empirical-publication inventory, leaving 32 empirical publications in Table S2. Two of these are state-only records with no content manipulation, leaving 30 content-bearing publications; 23 meet the baseline theory-addressed rule. Those 23 publications expand to 45 experiment-level rows because multi-experiment papers are partitioned at the experiment level. Yang et al. [161] (P26) and Sheth and Pham [162] (P27) are valenced publications but are not theory-addressed under the baseline rule. Gayet et al. [163] (P28) is likewise not baseline theory-addressed; its first experiment is valenced, whereas its second and third experiment rows are neutral visual controls. Baars [164] and Dehaene et al. [165] are theory/background sources rather than Table 2 inventory publications. Desender et al. [166,167] were legacy candidate records and are not part of the final Table S2 inventory under the revised content-bearing/theory-addressed definition.

At the experiment-row level, the same baseline contains 45 rows: 32 neutral visual, 4 neutral non-visual, 7 skeletal motor-conflict, 1 autonomic-effector motor-conflict, none valenced, and 1 interoceptive. The single autonomic-effector row belongs to Morsella et al. [65]; at publication level, that parent article is classified under its primary skeletal motor-conflict content, which is why Table 2 shows one autonomic experiment row but zero autonomic publications. The zero valence cell is fragile by design rather than absolute: under the most permissive no-theory-filter-plus-Whalen rule, valenced content rises to 6 of 57 experiment rows and 4 of 30 publications (Table S2 Sensitivity).

### 7.5. What Is Absent

The gaps identified here are not equivalent. M1 and M2 are sparsely occupied predictors: outside the supramodular family, the sampled trial-level accounts generally do not state predictions over policy count or incompatibility, so these coordinates do not presently discriminate among those silent theories. M5 is likewise an occupancy question rather than an adversarial result. The remaining genuinely opposed cell is M4c: SIT/passive-frame theory supplies a stated null for purely autonomic conflict, whereas the positive side is supplied only by INTERPRETED commitments for predictive-processing and higher-order accounts. Thus, even this adversarial cell depends asymmetrically on our interpretation of the opposing theories rather than on explicit positive statements by their proponents, and it should be treated as a candidate for proponent confirmation rather than a settled theory contrast.

Important empirical precursors therefore need to be acknowledged. Prestimulus heartbeat-evoked neural responses have been shown to predict subsequent visual detection, and the neural subjective frame literature proposes that cortical monitoring of visceral signals contributes to conscious perception [159,160]. These findings preclude the stronger claim that cortical–autonomic relations have not been measured in relation to access. The narrower unresolved question is whether a joint cross-channel coherence measure should function as a criterion governing access. Similarly, valenced and interoceptive content are not absent from consciousness research; the structured audit asks whether they occur in the theory-addressed sample under the stated inclusion rule.

### 7.6. Not a Technical Limitation

A gap reducible to unavailable methods is a difficulty, not a gap. Most of what the missing measurement requires is routine, and one component is not; we separate them, because the distinction decides which parts of the design in Section 9 are ready to run. Routine: policy conflict is manipulable and its subjective consequences measurable without overt movement [65]; report-independent access measures are established in the visual [14,153] and auditory [156] modalities; calibrated valenced stimuli [161] and standardised cardiac interoceptive measures [168] are off the shelf; and multisite preregistered adversarial designs are feasible at scale [1].

The absence is therefore partly theoretical and partly methodological. Where an account does not name policy count, incompatibility, or a joint cortical–autonomic coherence measure as a predictor, existing paradigms have little reason to manipulate that coordinate. At the same time, one component remains technically immature: there is no validated report-independent signature of access to interoceptive content comparable to established visual and auditory no-report paradigms. Cardiac interoceptive accuracy measures generally require report by construction. The autonomic-only adversarial condition should therefore be treated as a future validation problem rather than as an immediately executable discriminator.

This fixes the gap’s status: because both the predictor and the dependent variable are absent from existing designs rather than merely unanalysed, the gap cannot be closed by redescribing existing data. No reanalysis of the Cogitate dataset can recover an effect of policy conflict, since its stimuli—faces, objects, letters and false fonts under target detection—hold conflict load constant by construction [2].

### 7.7. Limitations of the Coding

Four limitations govern interpretation of the structured audit. First, the theory/account and empirical-study sets are purposive rather than exhaustive, so the tallies are not population prevalence estimates. Second, classification depends on source selection and on the boundary between explicit, interpreted, and not-located commitments; source loci and sensitivity analyses are therefore supplied. Third, M5 remains sensitive to how interpreted brain-body commitments are treated and is not used as an unqualified headline finding. Fourth, the independently generated ConTraSt scheme does not reproduce the submitted coordinates M1, M2, or M5 and therefore cannot validate them; it only demonstrates how occupancy changes under a different coordinate system. Independent second coding showed high pre-adjudication agreement for the theory-by-domain cells (115/120; 95.8%; Cohen’s κ = 0.930 on the five-category coding set) and complete agreement for the baseline Table 2 classifications at both publication (23/23) and experiment-row (45/45) levels. Lastly, because the second coder was a co-author familiar with the manuscript, these agreement statistics should be interpreted as internal consistency under a frozen codebook rather than as external inter-rater reliability.

## 8. Online Metarepresentation, Policy Candidacy, and Reconstruction

The upper part of the episode—registering one’s own processing, treating a proposed action as one option among several, and reconstructing it afterwards—contains three distinctions that are measured unevenly and are often conflated with neighbouring quantities. Section 8.1, Section 8.2, Section 8.3 and Section 8.4 separate what is already operationalised from what remains a proposed dissociation.

### 8.1. Precision, Not Registration

The case for treating self-registration as a separate quantity is not introspective: the accuracy of judgements about one’s own processing varies separately from that processing’s accuracy. Second-order signal detection supplies the apparatus, meta-d′ expressing metacognitive sensitivity in units of first-order d′ [169]; calibration, error sensitivity and performance dissociate, so “metacognition” unqualified means little [170]. Theta-burst TMS to prefrontal cortex impairs metacognitive awareness with discrimination intact [171]; lesion data implicate anterior prefrontal cortex, whose integrity predicts introspective rather than perceptual accuracy [172,173], with domain-general and domain-specific components [174] and a separate evaluative stage [175]. Registration itself grants no control. However, its precision predicts downstream change—confidence governs information search [176], poor metacognition accompanies resistance to counter-evidence [177], symptom dimensions shift metacognitive bias without touching performance [178]. The measurable variable is metarepresentational precision, not the fact of registration—a different claim from the one most theories in Table 1 make.

Two qualifications. Self-registration may not be self-directed: confidence compares contributions to joint decisions [179], is arguably a culturally acquired skill over implicit monitoring [180], secondary to mind-reading [181], and what a workspace needs to select what is admitted [182]; the radical version makes consciousness a learned metarepresentation [183,184,185]. And these claims rest on non-equivalent report scales: the Perceptual Awareness Scale exists because binary seen/unseen flattens intermediate states [186] and is more sensitive to weak contents than confidence [187], the discreteness dispute is unresolved [188] against workspace theories predicting a threshold [189], and scale and quantity are poorly coordinated [190].

### 8.2. Policy Candidacy

The second dissociation concerns policy candidacy (polyvariant choice)—the mode in which a proposed action policy ceases to be the sole command and becomes one member of a compared set. Three features define it: a change in the proposal’s status, a comparison set, and independence from both online metarepresentation and the success of control.

The name requires justification, because the obvious label is taken. In computational neuroscience, meta-control means arbitration between controllers and tuning of their parameters: model-based versus model-free learning by reliability [191], deciding how to decide [192], allocating effort by expected benefit [193], reviewed in [194]. That is parametric arbitration, not a policy represented as an object; since the term denotes the very neighbour this mode must be distinguished from, we do not use it, and this is its only appearance.

Existing paradigms measure everything adjacent to policy candidacy and not it. Meta-d′ measures the accuracy of evaluating a decision already taken [169]. The expected value of control measures the intensity of allocation in dorsal anterior cingulate cortex [195,196] over a cascaded prefrontal organisation [197] whose capacity limit is probably task separation, not fuel depletion [198]—the resource model having failed a preregistered replication [199] and multi-site test [200], effort recast as opportunity cost [201]. The stop-signal paradigm measures the speed of cancelling one prepared action [202,203]; its unconscious variant is decisive, masked stop signals recruiting frontal inhibitory machinery without awareness [204,205], and conscious and unconscious control engaging dissociable rather than graded mechanisms [206]. Conflict detection likewise has a canonical account in which the anterior cingulate recruits control with no commitment to consciousness [207]. The closest construct is intentional inhibition, where refusal is itself a choice [208,209]—but there too no alternative is generated and compared.

A correction here strengthens the case. The first of the three quantities operationalising policy candidacy—the size of the set under consideration—is already measured by option generation [210,211], including its neural basis [212], so the claim that none is measurable is too strong. Unmeasured are whether a decision depends on properties of the rejected alternatives, and whether metacognitive precision predicts option-set widening rather than correctness. The metric for the first, option-set widening, should be borrowed from option generation, not reinvented; the nearest precedent for the second is that the selection threshold shifts with urgency [54].

### 8.3. Report as Write-Back into the Self-Model

The third dissociation concerns what report is evidence for. That participants report not the causes of their behaviour but plausible theories of those causes remains established, if localised more precisely than originally [75], extending to introspection about current phenomenal content [213]. Choice blindness is the strong version: when already-chosen faces were switched, many switches went unnoticed and participants justified a choice they had not made [76], reproduced outside the laboratory [214].

The result that matters most is stronger than the standard reconstruction claim: switching participants’ own answers in a moral survey shifted the attitudes themselves, durably [215]. Reconstruction is therefore not a distortion of the record but a write-back into the model of oneself—report can become a cause of the subsequent state. The same holds for the sense of agency, an inference rather than access to the motor command [216,217], and for the construction of having chosen freely [218]. Two cautions: intentional binding [219] is contested, reproduced under illusory voluntary action [220] and sensitive to phenomenological control [221]; and the Libet result must be stated exactly—the averaged readiness potential [222] is not refuted as a measurement, its causal interpretation is, since stochastic accumulation makes it an artefact of averaging [223,224]. Hence “post hoc reconstruction” rather than “reflection”: the latter imports a transparency the data do not support, itself analysed as the unavailability of the model’s own modelhood to the system using it [162,163,164,165,166,167,225,226,227].

### 8.4. Outstanding Measurement Questions

The three dissociations differ in their present empirical maturity. Metarepresentational precision can already be tested with existing instruments; option-set generation is measurable, although whether decisions depend on properties of rejected alternatives remains less directly operationalised; and report-induced write-back into the self-model has one notable demonstration but little systematic follow-up. They therefore represent outstanding measurement questions rather than three wholly unmeasured phenomena.

## 9. Conclusions and an Executable Measurement Programme

The field’s problem is not a shortage of theories or of rigour, but a concentration of tests on a limited set of shared observables. The revised audit therefore distinguishes two opportunities: sparsely occupied coordinates that deserve measurement, and opposed predictions that can adjudicate among theories. M1 and M2 belong primarily to the first category; a positive effect there would establish empirical relevance but would not count against theories that are silent. The clearest adversarial leverage instead comes from conditions in which one account states a null or exclusion and another positively implicates the same domain.

One immediately executable programme is specified in Supplementary File S1. Study 1 holds policy count at three while varying incompatibility through whether three simultaneously licenced responses can be executed jointly across separate effectors or compete for a shared effector. Neutral-expression faces are presented near threshold; access is quantified with a preregistered no-report EEG index and a separate report-based perceptual-awareness measure, while ECG, electrodermal activity, respiration, and pupillometry provide concurrent autonomic measurements. The primary contrast tests incompatibility at fixed count, with the count branch retained as an estimation/control analysis rather than a competing-theory test.

The confirmatory sample is fixed at n= 120, which provides approximately 90% power for a paired effect of dz = 0.30 in the prespecified incompatibility contrast; the full trial budget, block structure, exclusions, analysis model, and power code are provided in Supplementary File S1 and the reproducibility deposit. Study 2 repeats the incompatibility manipulation with negative, neutral, and positive facial expressions. Its negative-versus-positive matched-arousal contrast is explicitly an author-derived hypothesis from the separate companion framework, not a prediction stated by any coded theory and not a direct theory test.

An effect of policy count or incompatibility would establish that the variable is empirically relevant under the controlled conditions of the experiment, but it would not by itself discriminate against GNWT or any other theory that makes no prediction over that coordinate. Direct adversarial interpretation is reserved for genuinely opposed commitments. At present the clearest candidate is the M4c autonomic-conflict boundary, but its positive side consists of our INTERPRETED readings of predictive-processing and higher-order accounts rather than explicit proponent statements; proponent confirmation would therefore be preferable before treating that cell as a definitive adversarial test.

The autonomic-only condition is therefore not presented as ready to run. Most homeostatic manipulations recruit skeletal effectors or intentions, which confounds a clean test of a somatic-only boundary, and existing interoceptive measures generally depend on report. The order of work is consequently pragmatic: the crossed incompatibility and valence studies use established visual access measures and can be executed now, whereas a future interoceptive validation study must first establish a report-independent access signature.

The deeper obstacle is the one named in Section 7.6: the third condition requires a report-independent measure of access to interoceptive content, and no such measure has been validated. This is why we present the third condition as the most discriminating test in principle and not as the one to run first. The order of work is the reverse of the order of interest—the crossed conflict and valence design uses established visual and auditory no-report measures and can be run now; the autonomic-only condition needs a validation study developing and calibrating an interoceptive no-report signature before it can adjudicate anything. Presenting it as ready would be the kind of overclaim this review has been criticising.

A closing note on what this review has and has not done. We have not proposed or tested a new theory here. We have mapped where sampled theories make explicit, interpreted, absent, or stated-null commitments; separated unoccupied coordinates from opposed predictions; and specified an experimental programme that turns neglected coordinates into measurements without claiming that silence constitutes falsification. A separate companion paper develops a theoretical account of some of these candidate coordinates; no claim from that framework is required for the conclusions of the present review.

## Figures and Tables

**Table 1:** Trial-level account summary across ten measurable domains. Y+ = explicit positive prediction; Y0 = explicit stated-null prediction; i+ = interpreted positive commitment; i0 = interpreted stated-null commitment; · = not located; NA = not applicable. Full source-level coding, including origin-level frameworks, is provided in Table S1.

M8	M7	M6	M5	M4c	M4b	M4a	M3	M2	M1	Account
Y+	i+	i+	·	·	·	·	·	·	·	AST
Y+	i+	·	·	·	·	·	i0	·	·	GNWT
Y+	Y+	·	·	i+	·	i+	i0	·	·	HOT
Y+	Y+	Y+	·	·	·	·	·	·	·	HOSS
Y+	·	Y+	·	·	·	·	·	·	·	IIT
i+	·	i+	i+	·	Y+	i0	i+	·	·	NSF
NA	·	·	·	Y0	·	i+	i0	i0	i0	PFT
i+	i+	i+	i+	i+	i+	Y+	Y+	·	·	PP
Y+	i0	·	·	·	·	·	·	·	·	RPT
i+	·	·	·	Y0	i0	Y+	i0	Y+	Y+	SIT

**Table 2:** Content classes in the revised theory-addressed, content-bearing sample. Counts are shown at publication and experiment-row levels; percentages are omitted because the sample is purposive.

Experiment Rows (n = 45)	Publications (n = 23)	Content Class
32	16	Neutral visual
4	2	Neutral non-visual
7	4	Motor conflict (skeletal)
1	0	Motor conflict (autonomic effector)
0	0	Valenced
1	1	Interoceptive
45	23	Total

## Data Availability

The complete coding dataset, study-level classifications, coding justifications, sensitivity analyses, and analysis scripts are available at https://doi.org/10.6084/m9.figshare.34043358 (accessed on 7 September 2026).

## Supplementary Materials

The following supporting information can be downloaded at: https://www.mdpi.com/article/10.3390/brainsci16101087/s1. Table S1 provides source-anchored theory-by-domain coding; Table S2 provides publication- and experiment-level classifications; Table S3 provides the ConTraSt-dimension robustness analysis; Table S4 contains the prediction register. Supplementary File S1 specifies the proposed experimental programme. The accompanying reproducibility deposit contains the frozen codebook, sensitivity analyses, and analysis scripts.
