# Supplementary material for: Beyond Cortical Signatures: Measurement Gaps Across Theories of Consciousness
Source: Brain Sci. 2026 Oct 9;16(10):1087. doi: 10.3390/brainsci16101087 (PMC13702712; doi:10.3390/brainsci16101087)
Supplement: Supplementary file 1 [file brainsci-16-01087-s001.zip › brainsci-4583950-supplementary file s1.pdf]

Supplementary File S1 — Proposed Experimental Programme

This protocol is referenced from Section 9 of the revised manuscript. Power figures are generated from the accompanying analysis files (power_study1.csv, power_study2.csv and power_mixed_check.csv) using section9_power.py; the cell list, block counts, session durations, success-rule power, retention probabilities and block orders are generated by protocol_numbers.py. Remaining author-set items are marked as [NUMBER: ...]. This is a proposed experimental protocol accompanying the review. Specifications marked 'to be preregistered' are prospective and have not yet been preregistered.

Protocol overview. Study 1 tests incompatibility at fixed policy count using one confirmatory no-report access contrast (C1a), alongside a separately preregistered report-based experienced-conflict contrast (CR). The count branch is treated as estimation rather than as a confirmatory theoretical test. Session-specific titration is completed before experimental blocks and remains fixed within blocks. The design uses seven presentation cells and 16 blocks per session, with effector mappings rotated across participants. Study 2 extends the same framework to valence.

S1.1 Design rationale

The design fixes the number of distinguishable responses at three and varies whether those responses can be executed jointly. This prevents incompatibility from being confounded with response-set size or simple congruency. The supramodular sources locate conscious conflict along the compatibility axis and allow compatible plans to co-occur without perturbing consciousness; the present manipulation therefore holds policy count constant while varying only whether the three demanded responses are mutually compatible.

S1.2 What is tested

Study 1 has one confirmatory contrast on the no-report access index, C1a, which tests P23. P23 is the authors' one-step derivation from supramodular interaction theory's stated P16: the same condition (incompatible intentions directed at the skeletomotor output system), a different outcome (access to the masked target that licenses them, instead of experienced conflict). The stated P16 is tested on its own outcome variable by a separately preregistered report-based contrast, CR, on the experienced-conflict rating collected in session 1 (register row P35). The count branch is estimation: C0 carries two author derivations with opposite signs, and P33 is a bounded estimate. The condition discriminates between no two theories.

Label	Prediction	Status	Source	Role in Study 1
P16	Conscious conflict when ≥ 2	stated (SIT)	Morsella 2005 (abstract); Morsella et	**tested on its own variable** by CR (row

Label	Prediction	Status	Source	Role in Study 1
	incompatible inclinations reach the skeletomotor output system; outcome variable experienced conflict; nothing stated beyond two		al. 2016 §2.4	P35): the experienced-conflict rating at 0 against 1 and 3 shared effector pairs, count 3, session-1 probe trials, after the response
P23	Access to a masked target rises with the incompatibility of the policies it licenses, at fixed count and load	derived by the authors in one step from P16 (same condition, different outcome); not endorsed by SIT proponents	this manuscript	**the confirmatory contrast C1a** (EEG-index and PAS-report versions)
P32	Access is higher when ≥ 2 co-executable policies are in force than when one is	derived by the authors from P16 with two auxiliary assumptions	this manuscript	estimation: the sign of C0 (positive)
P25	More competing policies degrade access (load)	derived; GNWT plus a capacity assumption GNWT has not made	this manuscript	estimation: the sign of C0 (negative)
P33	No further increase in access from 2 to 3 co-executable policies	derived by the authors; the source is silent beyond two, and silence is not a stated null	this manuscript	bounded estimate C0-eq: the equivalence bound within which the 3 – 2 difference falls is reported

Label	Prediction	Status	Source	Role in Study 1
—	Cortical–autonomic coupling index	estimation only; no coded theory predicts over it	—	estimated with interval

Why the count branch is an estimate and not a test. P32 and P25 predict opposite signs on C0, and neither is stated by its source; a confirmatory test would have to pick one. The branch is kept because a nuisance account — more levers in force give more access — predicts a positive C0 (one lever at count 1, three at count 3) and a negative C1a (three levers at 0 shared pairs, one at 3 shared pairs), the opposite of P23; the C0 estimate therefore tells the reader whether the C1a result can be attributed to the number of levers. A null on C1a constrains the authors' derivation within the preregistered bound; it does not refute the theory, whose stated outcome variable is experienced conflict and which CR tests directly.

S1.3 Participants

Healthy right-handed adults aged 18–40, with normal or corrected visual acuity and colour vision, and no neurological, psychiatric or cardiovascular history or related medication. Confirmatory sample N = 120 (119 required for power 0.90 at dz = 0.30 for the single uncorrected contrast C1a, rounded up to a multiple of the six lever rotations), fixed in advance; no sequential monitoring. The power of the two-version access-index rule at this N is given in S5.13. Recruitment continues until 120 participants have complete, non-excluded data for both sessions; the number recruited and the number excluded on each criterion are reported as k of n.

S1.4 Stimuli

Targets are grey-scale neutral-expression faces of six identities (three female) from the Karolinska Directed Emotional Faces set [1], equated on luminance histogram and spatial-frequency spectrum with the SHINE toolbox [2], cropped to an oval and shown at 4°. Each face carries three task features orthogonal to identity: isoluminant tint (three hues), position (above, at or below fixation) and aperture shape (circle, square, diamond). Each rule maps its three feature values onto the three positions of a lever (up, middle, down). The stimulus set is restricted to feature conjunctions for which the three demanded lever positions are mutually distinct, so that on every trial the target demands three distinguishable responses; the six permutations of (up, middle, down) over the three rules occur equally often. Rule-to-feature and value-to-position assignments are rotated across participants.

S1.5 Effectors and the incompatibility manipulation

Three three-position levers: left hand, right hand, dominant foot. A response is a lever position. Two responses on different levers can be executed jointly; two

responses on the same lever cannot. Incompatibility on a trial is the number of pairs of demanded responses that share a lever:

Shared pairs	Effector mapping in force	Co-executable?
0	hue → lever A, position → lever B, shape → lever C	all three jointly
1	two rules on one lever, the third on another	the pair on the shared lever cannot be executed jointly
3	all three rules on one lever	none jointly

The mapping is fixed for a block, announced by a block cue at block start (a diagram of the three levers with the rule symbols placed on them, shown for 5 s and available on request during the inter-block break) and overlearned in training; the stimulus set is identical across mappings. The count branch keeps all rules on separate levers (0 shared pairs) and puts one, two or three rules in force.

Effector identity is rotated within participant for the 1-shared cell and between participants elsewhere. In the 3-shared cell all rules sit on one lever, whereas in the 0-shared cells they sit on three. Each participant completes the three 1-shared presentation cells, one for each pair of rules that shares a lever, with the shared lever rotated between participants. The single 3-shared cell's lever, the rule-to-lever assignment of the 0-shared cells, the lever of the count-1 cell and the lever pair of the count-2 cell are rotated between participants in six orders. This balances effector identity across participants in every analysis cell; any residual lever effect is reported as an exploratory factor.

S1.6 Cell list and block scheme (from protocol_numbers.json and block_orders_study1.csv)

Seven presentation cells, collapsing to five analysis cells. The cell list, stated explicitly:

Presentation cell	Analysis cell	Blocks per session	What is balanced, and how
count 1, 0 shared	count 1, 0 shared	2	one rule on one lever; lever rotated between participants
count 2, 0 shared	count 2, 0 shared	2	two rules on two levers; lever pair rotated between participants
count 3, 0 shared	count 3, 0 shared (C1a cell)	**3**	three rules on three levers; rule-

Presentation cell	Analysis cell	Blocks per session	What is balanced, and how
			to-lever assignment rotated between participants
count 3, 1 shared, pair A	count 3, 1 shared (C1a cell, pooled)	2	rules 1 and 2 share a lever; shared lever rotated between participants
count 3, 1 shared, pair B	count 3, 1 shared (C1a cell, pooled)	2	rules 1 and 3 share a lever; shared lever rotated between participants
count 3, 1 shared, pair C	count 3, 1 shared (C1a cell, pooled)	2	rules 2 and 3 share a lever; shared lever rotated between participants
count 3, 3 shared	count 3, 3 shared (C1a cell)	**3**	all rules on one lever; lever rotated between participants

Blocks (to be preregistered). Presentation cells are blocked, not interleaved: a block contains trials of one presentation cell under one fixed mapping. Each session runs 16 blocks: two per presentation cell and three in the two single-cell C1a cells (count 3 at 0 shared and at 3 shared). In session 2 a block has 52 trials (13 probe, 39 no-probe); in session 1 a block has 32 trials (8 probe, 24 no-probe). The third block does not equalise precision between the single C1a cells and the pooled 1-shared cell: the single cells reach about 99 usable no-report trials in session 2 against about 199 in the pooled cell (S5.8); the third block removes the risk that a single cell falls below the usable-trial floor, and it roughly halves the gap in usable trials (the single-to-pooled ratio rises from a third to a half) — no more than that.

Block order for unequal block counts (to be preregistered; block_orders_study1.csv). A session's 16 blocks are a row of a cyclic 7 × 7 Latin square over the seven presentation cells (blocks 1–7), then the two extra C1a blocks at the session midpoint (blocks 8–9; count 3 at 0 shared and at 3 shared, in either order), then the same row reversed (blocks 10–16). Seven rows × two orders of the midpoint pair give 14 sequences; session 2 uses the next row of the square with the midpoint pair swapped. The scheme is checked by protocol_numbers.py: every cell appears once in blocks 1–7 and once in blocks 10–16, and the mean block position is

8.5 for every cell, so cell and time-in-session are not confounded in the mean. In 4 of the 14 sequences the Latin row ends in a C1a cell, whose third block then follows its second across one break under an unchanged mapping; this is reported, not avoided. Sequence and lever rotation are assigned from two lists fixed at preregistration (participant index modulo 14 and modulo 6); at N = 120 each sequence is used 8 or 9 times and the realised counts are reported. Each block begins with the block cue of S5.5 and, in session 1, with four full-contrast practice trials under the new mapping that are not analysed. Because the mapping changes only at block boundaries, a participant never switches mapping within a block; the block-wise cross-validation of S5.10 respects the block structure.

S1.7 Trial structure, probe, ratings and sessions

Fixation 500–800 ms; target 33 ms at the contrast frozen in the session's titration phase; pattern mask at 50 ms SOA for 200 ms [3]; 1,000 ms blank; then either the probe phase or nothing; then an inter-trial interval of 2.5–3.5 s, jittered uniformly (design choices; the interval is set by the autonomic windows of S5.11). The planning value is 6 s per trial without a rating, 8 s with the Perceptual Awareness Scale rating and 9 s with the experienced-conflict rating.

Titration phase (to be preregistered). Each session opens with a separate titration phase of 48 trials (a design choice, to be replaced by the pilot's staircase length) in which a staircase on the Perceptual Awareness Scale rating [4] sets the target contrast to a visibility of about one half; the contrast is then frozen for all blocks of that session, nothing adapts within blocks, and the difference between the two sessions' frozen contrasts is reported descriptively as the drift over the 48 h or more between sessions. Session 2 is titrated afresh because the contrast would drift; its blocks remain no-report.

Probe. On a quarter of trials a probe (the symbol of one rule, 1,000 ms) names one rule in force and the participant executes that rule alone on the appropriate lever, or gives a fourth response (a foot-pedal press) for "no face"; on three quarters of trials nothing is required. The probe names one rule and one response; joint execution of several responses is never required. Probe trials keep the rules active across the block and supply the manipulation checks. Open question, stated plainly: the design cannot establish that a participant forms three incompatible intentions at target onset rather than holding three rules in memory until the probe selects one. Two checks bear on it. Probe-response latency as a function of shared pairs (0, 1, 3) at count 3 is reported with estimate and interval and is not a confirmatory test. The experienced-conflict rating below is the second, and the theory's own: if incompatible intentions are formed, the rating must rise with shared pairs.

Experienced-conflict rating (session 1, probe trials only, after the response). On every probe trial of session 1, after the probe response has been given — never before, since a rating before the response would disrupt rule execution and the latency measure — the participant answers "How strong was your urge to make a mistake?" on the 8-point scale of Morsella et al. 2009 [15] (1 "almost no urge", 8

“extremely strong urge”), the outcome variable in which P16 is stated. The rating serves two purposes: (a) the separately preregistered report-based contrast CR (S5.12), a direct test of the stated P16 on its own variable; (b) the numbered pilot stop criterion of S5.14. The rating is not collected in session 2, whose blocks are no-report.

Sessions. Session 1: titration phase, training to criterion on the mappings, then the 16 blocks with a Perceptual Awareness Scale rating on every no-probe trial (the classifier labels and the PAS-report version of C1a; the rating does not feed the staircase) and the experienced-conflict rating on every probe trial after the response; the data on which the classifier is trained. Session 2: titration phase, then the same 16 blocks without any rating; only session-2 no-probe trials enter the confirmatory no-report estimate. Two sessions at least 48 h apart.

S1.8 Presented and usable trials (from protocol_numbers.json)

The confirmatory no-report estimate uses session-2 no-probe trials only. Budget parameters: probe fraction one quarter, assumed loss rate 0.15, target ≥ 64 usable no-report trials per analysis cell. The smallest two-block presentation count with an integer number of probe trials per block whose no-probe trials survive the loss rate at an expected 64 is 104 (raw minimum 100.39), which fixes the session-2 block at 52 trials; a three-block cell therefore presents 156.

Presentation cell	Blocks	Session 2 presented (probe + no-probe)	Expected usable no-report	Session 1 presented	PAS-rated after loss	Experienced-conflict ratings (probe trials)
count 1, 0 shared	2	104 = 26 + 78	66.3	64	40.8	16
count 2, 0 shared	2	104 = 26 + 78	66.3	64	40.8	16
count 3, 0 shared	3	156 = 39 + 117	99.45	96	61.2	24
count 3, 1 shared (three cells pooled)	3 × 2	312 = 78 + 234	198.9	192	122.4	48
count 3, 3 shared	3	156 = 39 + 117	99.45	96	61.2	24
**Session totals**	**16**	**832 = 208 + 624**		**512**	**384 rated**	**128**

The mixed-model check of S5.13 is calibrated to the floor of 64 usable trials per analysis cell and so is conservative for every C1a cell. The 99.45 and 66.3 figures are expectations, not guarantees. Under a simple model of independent trial loss at the planning rate the probability that a three-block C1a cell (117 no-probe trials) reaches 64 usable trials is greater than 0.9999, and so is the joint probability for the two single C1a cells; for a two-block count cell (78 no-probe trials) it is 0.8151, and 0.6644 for the two count cells jointly (protocol_numbers.json; illustrative, since artefact losses are correlated within a session). In the two single C1a cells had two blocks each and the joint probability of 0.6644 applied to the confirmatory cells; the third block moves that risk off the confirmatory contrast. For the count cells, which are estimation, two provisions are preregistered: (i) a count cell that ends session 2 below the floor is completed by one repeated block of that cell at the end of the session (a fixed rule, not a judgement); (ii) a participant whose count cell is still below the floor after the repeat is retained for C1a and flagged in the count estimate, which is reported with and without flagged participants. A reserve calculation: the smallest two-block budget that gives a two-block cell a probability of at least 0.95 of reaching the floor is 112 presented (probability 0.9887); it is not the plan.

Duration. Session 2: titration 48 trials × 8 s = 6.4 min; 832 trials × 6 s = 83.2 min; 15 inter-block breaks of 1 min; 104.6 min in all, plus set-up. Session 1: titration 6.4 min; 384 PAS-rated no-probe trials × 8 s and 128 rated probe trials × 9 s = 70.4 min; 64 practice trials × 6 s = 6.4 min; 15 min of breaks; 98.2 min in all, plus training to criterion and set-up, for which [NUMBER: set-up, titration and training time — to be measured in the pilot].

S1.9 Exclusions (to be preregistered)

Accuracy below 0.90 on full-contrast probe trials after training; frozen visibility outside 0.35–0.65 on the Perceptual Awareness Scale at the end of either session's titration phase; cross-validated classifier AUC below 0.70 in session 1 (S5.10, S5.14); more than 0.30 of trials lost to artefact in either session; fewer than 64 usable no-report trials in any of the three C1a analysis cells of session 2 (count 3 at 0, 1 and 3 shared pairs; thresholds s as design choices, the last restricted to the confirmatory cells in current protocol — count cells below the floor follow S5.8). A participant excluded on any criterion is replaced until N = 120 is reached. The AUC criterion defines the population to which the confirmatory result applies: adults in whom awareness of a masked face is decodable from posterior EEG at AUC ≥ 0.70 under these conditions. The proportion excluded on this criterion is reported as k of n recruited, and the confirmatory conclusion is stated for this population only.

S1.10 The access index (specification to be frozen)

The access index is the trial-wise decoded evidence (logit) for "seen" from a per-participant classifier trained on session-1 no-probe trials labelled by the Perceptual Awareness Scale rating. Every element below is a design choice to be preregistered; the pilot (S5.14) may adjust them before preregistration and nothing may change after it.

- Channels. 64-channel EEG in the extended 10–20 layout, 0.1–40 Hz, 500 Hz sampling, average reference after cleaning. The classifier uses the 17 parieto-occipital channels P7, P5, P3, P1, Pz, P2, P4, P6, P8, PO7, PO3, POz, PO4, PO8, O1, Oz, O2. No frontal or central channel enters the index, so that a fronto-central report-related component cannot carry it.
- Preprocessing. Bad-channel detection and spherical-spline interpolation; independent component analysis with automated labelling and removal of ocular and cardiac components; epochs −200 to 800 ms around target onset, baseline −200 to 0 ms; rejection of epochs exceeding ±100 µV on any retained channel; downsampling to 100 Hz. The same pipeline, with parameters frozen from session 1, is applied to session 2 without re-fitting.
- Features and classifier. Mean amplitude in twenty 20-ms bins from 100 to 500 ms post-target on the 17 channels (340 features), a window that covers the visual awareness negativity, which survives removal of report, and is cut at 500 ms to limit the contribution of the report-dependent P3b, which it cannot exclude entirely [5–8]. Classifier family: linear discriminant analysis with shrinkage regularisation of the covariance [18]. No other family is tried.
- Labels. The rating is binarised as 1 (“no experience”) against 2–4 (any experience); the binarisation is fixed and is not tuned to maximise separation. Session-1 no-probe trials supply 384 labelled trials per participant before loss (S5.8).
- Validation and leakage control. Nested cross-validation on session 1 with block-wise folds: the outer loop leaves one of the 16 session-1 blocks out, so that no trial from the same block (same mapping, same time in session) appears in both training and test; the inner loop selects the shrinkage parameter on the training blocks only. Stimulus identity is balanced across folds. The reported session-1 AUC is the outer-fold average.
- Transfer condition (index of seeing, not of conflict). Trained on the blocks of one incompatibility level, the classifier must reach AUC ≥ 0.70 on the blocks of each other level. Rating-based cell differences are reported alongside index-based ones descriptively; reproducing them is not a criterion (S5.14).
- Freezing. After session 1 the shrinkage parameter, the channel set, the bins, the preprocessing parameters and the per-participant weights are written to the archive with a hash before any session-2 trial is scored; the frozen files are part of the deposit. The index is EEG-only: no pupil, blink or oculomotor feature enters it, so that the autonomic channels of S5.11 remain independent of it. The session-2 contrast is frozen separately, at the end of the session-2 titration phase (S5.7); the classifier is not re-fitted to it, and the session-2 decoded evidence is reported alongside the session-2 frozen contrast.

The index is a candidate marker of access and not a measure of M5. C1a is also computed on the session-1 Perceptual Awareness Scale rating (the PAS-report version); the success rule of S5.12 requires both versions.

S1.11 Autonomic channels and the coupling metric

Lead-II ECG, skin conductance and pupil diameter are recorded throughout both sessions alongside the EEG. Timing markers are written to all channels at fixation onset, target onset, probe onset, response, rating onset and inter-trial-interval onset. The inter-trial interval of 2.5–3.5 s (S5.7) is set so that the response windows below are complete before the next fixation on most trials, but at a planning value of 6 s per trial the skin-conductance response to one target overlaps the next trial; overlapping responses are therefore separated by deconvolution rather than by lengthening the interval.

- Heartbeat-evoked potential. Mean fronto-central amplitude 250–400 ms after the last R-peak preceding the target by at least 400 ms, cardiac-field artefact controlled by surrogate-R-peak permutation; direction two-sided [9,10]; cardiac phase at onset as covariate.
- Skin conductance. Phasic driver recovered by continuous decomposition analysis [11,12], which models the overlapping responses of successive trials; the trial response is the integrated driver 1–5 s after target onset.
- Pupil. Baseline-corrected mean diameter 0.5–2.5 s after target onset, with a general-linear-model deconvolution of the pupil trace using target onsets, probe onsets and rating onsets as regressors to separate the response to the current target from the tail of the previous one, from the probe and from the rating [13].

Coupling index: (a) the per-participant, per-cell standardised slope of decoded access on pre-stimulus heartbeat-evoked amplitude; (b) the trial-wise correlation of decoded access with the phasic skin-conductance and pupil responses. Both are reported with point estimate and 0.95 interval per cell and as a function of shared pairs. Estimation only: no coded theory predicts over the index, it is not a measure of M5 in the organism-wide sense of §7, and no hypothesis test is attached to it.

S1.12 Contrasts, multiplicity and the success rule

Effect sizes are dz on per-participant contrast scores of the cell-mean outcome; α = 0.05 two-sided.

Contrast	Outcome	Cells (order)	Weights	Test	Hypothesis	Status
C1a	access index (session 2); PAS rating (session 1)	shared 0, 1, 3 at count 3	−1, 0.5, 0.5	paired t	P23 (one-step derivation from P16)	**confirmatory** — the only confirmatory contrast on access
CR	experienc	shared 0,	−1, 0.5,	paired t	P16	**confirma**

Contrast	Outcome	Cells (order)	Weights	Test	Hypothesis	Status
	ed-conflict rating (session 1, probe trials)	1, 3 at count 3	0.5		(stated; row P35)	**tory, report-based**, separately preregistered
C0	access index; PAS rating	count 1, 2, 3 at 0 shared	−1, 0.5, 0.5	estimate with 0.95 interval, two-sided	P32 (positive) against P25 (negative)	estimation
C0-eq	access index; PAS rating	count 2, 3 at 0 shared	0, −1, 1	TOST bound reported [14]	P33	bounded estimate
C1b-lin	access index	shared 0, 1, 3 at count 3	−1.3333, −0.3333, 1.6667 (linear in shared pairs)	paired t	—	exploratory
C1b-omni	access index	shared 0, 1, 3 at count 3	two orthogonal contrasts	Hotelling T^2, $F(2, N - 2)$	—	exploratory

Multiplicity (to be preregistered). There is one confirmatory contrast on the access index, C1a, tested at $\alpha = 0.05$ without correction; the Holm procedure of [19] is withdrawn with the second contrast. CR is a separately preregistered confirmatory test of a different hypothesis (the stated P16) on a different outcome variable (the experienced-conflict rating), in its own family at $\alpha = 0.05$; the two tests do not support a common claim, so no correction is applied across them, and both p-values are reported so that a reader who prefers a joint family can apply one. C0 and C0-eq are estimates: C0 is reported two-sided with its interval and the sign is read against P32 and P25; C0-eq reports the narrowest symmetric bound (in dz) within which the count-3 minus count-2 difference is equivalent to zero by two one-sided tests at $\alpha = 0.05$ each, and P33 is supported only if that bound is ±0.30 dz or narrower — a bound reported, not a confirmatory test. C1b is exploratory and is not reported as a test of any hypothesis. Ordinal weights (−1, 0, +1) are not used for C1b because they would treat the step from 1 to 3 shared pairs as equal to the step from 0 to 1. A null on C1a is interpreted through a TOST with the same ±0.30 dz bound.

Success rule (to be preregistered). C1a is counted as supported only if (i) its EEG-index version is significant in the direction predicted — an increase of access with incompatibility — and (ii) its PAS-report version, computed on the session-1 ratings (61.2 rated trials expected in each single C1a cell and 122.4 in the pooled cell after loss, fewer than the 99.45 and 198.9 no-report trials of session 2, so this version is the less powerful of the two), is significant with the same, predicted, sign. A significant effect in the opposite direction is a disconfirmation, not a pass. A contrast that passes on one version only is reported as unsupported, with both estimates. CR is judged on its own: significant in the predicted direction (rating rises with shared pairs) or not. The two-version rule is conjunctive and its power is lower than the power of either version alone (S5.13).

S1.13 Power

C1a (from power_study1.csv). Paired-t sample sizes, uncorrected α = 0.05:

dz	N for power 0.80	N for power 0.90
0.20	199	265
0.30	90	119
0.40	52	68

Fixed sample N = 120: achieved power 0.9032 at dz = 0.30 for C1a, uncorrected; minimum detectable dz at power 0.90 is 0.2983.

Two-version rule (from protocol_numbers.json). The EEG-index and PAS-report versions must both pass. Their joint power cannot be derived analytically without the correlation between the two versions, which is unknown before the pilot; under independence it is the product $0.9032^2 = 0.8157$, and the PAS version, resting on the smaller session-1 trial count, is treated here as equally powered, which overstates the rule's power by an amount the pilot will show. For power 0.90 under the rule and independence a single contrast would need N = 146. The two versions are measured on the same participants and stimuli and are expected to be positively dependent, which would raise the joint figure above the product, but by an amount [to be computed from the pilot correlation]. The fixed N = 120 is therefore adequately powered for C1a and short of 0.90 for the two-version rule under independence; the rule is retained because it protects against a decoder artefact, and its cost in power is stated rather than hidden.

CR, the report-based test of P16 (from power_study1.csv). Power is computed from the same N = 120 at an assumed effect size, since no prior study has measured the experienced-conflict rating under a block-wise effector mapping with masked targets. The assumption is dz = 0.50, and it is an assumption: the only anchor, incompatible against compatible intentions on the same rating in Morsella et al. 2009, t(13) = 6.21 [15], corresponds to dz = 1.660, an upper bound obtained with visible stimuli and trial-wise incompatibility, and the assumed value is less than a third of it. At dz = 0.50 the power of CR at N = 120 is 0.9997; at dz = 0.30 it is 0.9032,

at dz = 0.20 it is 0.5844. CR rests on 24 rated probe trials per single C1a cell and 48 in the pooled cell in session 1 (S5.8), so its per-participant measurement noise is larger than that of C1a; the assumed dz is defined at the participant level and already includes that noise.

C0 and P33 (estimation). For orientation only, a paired t on C0 at N = 120 would have power 0.9032 at dz = 0.30. For C0-eq at N = 120 and a true effect of zero, the probability of establishing equivalence within a bound is 0.406 for ±0.20 dz, 0.895 (Monte Carlo 0.896) for ±0.30 and 0.993 for ±0.40; the N that would give 0.90 is 272, 122 and 70. These figures describe how informative the reported bound is expected to be; no test is attached. The ±0.30 bound is the planning effect size itself, stated in units of the population SD; it is not to be replaced by a fraction of the SD estimated on the final data.

Exploratory C1b at N = 120 (uncorrected): linear-in-shared-pairs power 0.5844 (dz 0.20), 0.9032 (0.30), 0.9915 (0.40); N for 0.90 is 265 / 119 / 68. Two-df omnibus power 0.4762 / 0.8359 / 0.979; N for 0.90 is 320 / 144 / 83.

Mixed-model check (power_mixed_check.csv). At N = 120 and 64 usable trials per cell (the calibration floor; the session-2 budget expects 99.45 in the single C1a cells and 198.9 in the pooled cell), 200 simulated data sets calibrated to dz = 0.30 (statsmodels 0.14.6; power_mixed_check.csv from the full run, seed 20260924) — C1a: analytic 0.903, two-stage paired t 0.935, linear mixed model with random slopes 0.935 (Monte Carlo SE about 0.021), 0 non-converged; C0: 0.903 / 0.900 / 0.900, 0 non-converged. Agreement with the analytic value is expected by construction; the check confirms the calibration and is not an independent estimate. A run of reproduce.py with --skip-mixed writes a single row skipped to this file and never reports the check as done. The two-df omnibus power assumes the whole effect lies along one contrast direction.

S1.14 Mandatory pilot

A pilot of [NUMBER: pilot sample size — to be set by the authors] participants runs the full session-1 procedure and a shortened session 2 of [NUMBER: blocks]. Four criteria, each to be preregistered, must all be met before the confirmatory sample begins:

1. Threshold. The titration phase brings Perceptual Awareness Scale visibility inside 0.35–0.65 for every included pilot participant, and the frozen contrast holds visibility inside that band across the blocks of the session (reported per block).
2. Measurement validity. The classifier specified in S5.10, trained on the blocks of one incompatibility level, reaches cross-validated AUC ≥ 0.70 on the blocks of each of the other two levels, in at least [NUMBER: k of n pilot participants]. This criterion tests whether the index measures seeing rather than conflict; it does not require the index to reproduce any cell difference, so it cannot select participants for the hypothesised effect. It does select participants

whose awareness is decodable at this accuracy, and that selected population is the one named in S5.9 to which the confirmatory result applies.

3. Accuracy on the probed rule. Full-contrast accuracy on the single probed rule at least 0.90 in every presentation cell, including the 3-shared cell, in which the probed rule must be executed alone on the shared lever. Joint execution is not required by the probe and is not a criterion. The pilot also reports probe-response latency by shared-pairs level (S5.7) descriptively.
4. The manipulation forms incompatible intentions (numbered stop criterion). On the session-1 experienced-conflict ratings, the standardised mean difference between the 3-shared and the 0-shared cell at count 3 (dz on per-participant differences over the pilot sample) must be at least 0.50. Below 0.50 the effector mapping is not producing experienced incompatibility at the rate the theory requires — intentions are not being formed — and the study stops and is redesigned. The number is justified in one sentence: 0.50 is the effect size assumed for the confirmatory rating test CR (S5.13), so a pilot effect below it would leave that test unwarranted at the planned N, and it is less than a third of the anchor of Morsella et al. 2009 (dz = 1.660) [15], so a manipulation that fails to reach it is failing, not merely weaker than the anchor. The pilot dz is reported with its interval whether or not the criterion is met.

If any criterion fails the study stops and the pilot is reported. If the probe fraction, the loss rate, the staircase length or the trial duration observed in the pilot differs from the planning values, the presented-trial counts and session durations are re-derived by section9_power.py and protocol_numbers.py before preregistration.

S1.15 Study 2 — valence (Supplementary only)

Rationale and status. Study 2 asks whether valenced content shifts the access threshold at matched arousal, the cell that Table 2 shows to be empty in the checked theory-addressed sample, and whether the incompatibility effect of Study 1 differs by valence. The first question is a confirmatory test of P34, an author-derived hypothesis. It is not a test of the stated P13: P13 (Seth 2013; Joffily & Coricelli 2013) concerns experienced valence and its relation to the rate of change of free energy, and says nothing about the direction of access differences between sad and happy faces at matched arousal. Deriving P34 from P13 needs three auxiliary assumptions, listed in the P34 register row, none of which the source states; a result on P34 corroborates or constrains the authors' derivation and not predictive processing. The second question is estimation.

Stimuli. The Study 1 identities in sad, neutral and happy expressions from the same set, whose validation supplies hit-rate, intensity and arousal norms [1]. Sad rather than fearful expressions are used because threat gains access through routes that need not involve valence [16,17]. Sad and happy exemplars are matched on normative arousal; neutral exemplars are necessarily lower, so the critical contrast is negative against positive at matched arousal. Equated with SHINE as in Study 1.

Pupil and skin conductance: manipulation checks, covariates only with justification. The trial-wise pupil and skin-conductance responses serve first as manipulation checks that the normative arousal matching holds in these participants: a difference in either between sad and happy trials is reported and, if present, the matching has failed for that participant, who is [to be decided at preregistration: excluded or flagged]. They are not entered as covariates in the primary analysis, because a valenced face may change pupil size and skin conductance as a consequence of the manipulation itself; adjusting for a consequence of the manipulation would remove part of the effect under test. They enter only a sensitivity analysis, reported alongside the primary one, whose purpose is to show how much of any negative − positive difference survives adjustment for residual arousal.

Design (from protocol_numbers.json). Nine cells: valence (negative, neutral, positive) × shared pairs (0, 1, 3) at count 3 (the count branch is not repeated), two blocks each, 18 blocks per session; titration phase, probe, ratings and sessions as in Study 1 — session 1 with 64 presented trials per cell (576 trials; about 109.8 min with titration, practice and breaks) and session 2 with 104 per cell (936 trials; about 117.0 min with titration and breaks), 1512 presented trials per participant in all. Session 2 of Study 2 is longer than Study 1's and is reviewed in the pilot. Access index, autonomic channels and exclusions as in Study 1, with one added exclusion — a participant whose own post-session ratings fail to order the faces negative < neutral < positive is excluded.

Sample and power (from power_study2.csv and protocol_numbers.json). Confirmatory contrast: negative − positive at matched arousal, paired t, dz = 0.30 → N = 119 at power 0.90 (0.80: 90); dz = 0.20 → 265 (0.80: 199); dz = 0.40 → 68 (0.80: 52). Study 2 sample N = 120 (119 required; rounded to a multiple of the six lever rotations, as in Study 1), fixed, no sequential monitoring; power for the single contrast at that N is 0.9032 uncorrected (one confirmatory contrast, so no multiplicity correction is needed), and under the two-version rule the power is 0.8157 if the two versions were independent (see S5.13 for the status of that assumption). Incompatibility × valence interaction (contrast-of-contrasts): a confirmatory test at dz = 0.20 would need N = 265; at N = 120 its power is 0.584 at dz = 0.20 and 0.903 at dz = 0.30. The interaction is therefore estimated and reported with its estimate and interval, not tested. Minimum detectable dz for any paired contrast at N = 120 is 0.2983.

Interpretation. A negative − positive effect corroborates the authors' derivation P34 from the one trial-level account that predicts over M3, and separates none of the coded theories: GNWT, higher-order thought theory, supramodular interaction theory and passive frame theory carry constructed nulls over valence that none has stated. The interaction estimate will be reported for any theory that later states a prediction over it.

S1.16 Data, code and deposit

section9_power.py (v2.4) writes power_study1.csv, power_study2.csv, trials_budget.csv, trials_budget_cells.csv, trials_budget_analysis_cells.csv, power_mixed_check.csv and section9_key_numbers.json; protocol_numbers.py (v3.3) writes protocol_numbers.json (cells, blocks, session durations, two-version-rule power, the report-based test, the pilot stop threshold, retention probabilities) and block_orders_study1.csv (the 14 block sequences with their balance checks); reproduce.py (v2.3) re-derives every number in this protocol and in §9.1 that comes from those scripts and checks the outputs against expected_hashes.json. The index specification, effector-mapping tables, block orders, power outputs, and analysis scripts are included in the accompanying reproducibility deposit.

References (File S1)

1. Goeleven, E.; De Raedt, R.; Leyman, L.; Verschuere, B. The Karolinska Directed Emotional Faces: A validation study. Cognition & Emotion 2008, 22, 1094–1118. https://doi.org/10.1080/02699930701626582
2. Willenbockel, V.; Sadr, J.; Fiset, D.; Horne, G.O.; Gosselin, F.; Tanaka, J.W. Controlling low-level image properties: The SHINE toolbox. Behavior Research Methods 2010, 42, 671–684. https://doi.org/10.3758/brm.42.3.671
3. Del Cul, A.; Baillet, S.; Dehaene, S. Brain Dynamics Underlying the Nonlinear Threshold for Access to Consciousness. PLoS Biology 2007, 5, e260. https://doi.org/10.1371/journal.pbio.0050260
4. Ramsøy, T.Z.; Overgaard, M. Introspection and subliminal perception. Phenomenology and the Cognitive Sciences 2004, 3, 1–23. https://doi.org/10.1023/b:phen.0000041900.30172.e8
5. Pitts, M.A.; Metzler, S.; Hillyard, S.A. Isolating neural correlates of conscious perception from neural correlates of reporting one's perception. Frontiers in Psychology 2014, 5. https://doi.org/10.3389/fpsyg.2014.01078
6. Cohen, M.A.; Ortego, K.; Kyroudis, A.; Pitts, M. Distinguishing the Neural Correlates of Perceptual Awareness and Postperceptual Processing. The Journal of Neuroscience 2020, 40, 4925–4935. https://doi.org/10.1523/jneurosci.0120-20.2020
7. Schlossmacher, I.; Dellert, T.; Pitts, M.; Bruchmann, M.; Straube, T. Differential Effects of Awareness and Task Relevance on Early and Late ERPs in a No-Report Visual Oddball Paradigm. The Journal of Neuroscience 2020, 40, 2906–2913. https://doi.org/10.1523/jneurosci.2077-19.2020
8. Dellert, T.; Müller-Bardorff, M.; Schlossmacher, I.; Pitts, M.; Hofmann, D.; Bruchmann, M.; Straube, T. Dissociating the Neural Correlates of Consciousness and Task Relevance in Face Perception Using Simultaneous EEG-fMRI. The Journal of Neuroscience 2021, 41, 7864–7875. https://doi.org/10.1523/jneurosci.2799-20.2021

9. Park, H.D.; Correia, S.; Ducorps, A.; Tallon-Baudry, C. Spontaneous fluctuations in neural responses to heartbeats predict visual detection. Nature Neuroscience 2014, 17, 612–618. https://doi.org/10.1038/nn.3671
10. Al, E.; Iliopoulos, F.; Forschack, N.; Nierhaus, T.; Grund, M.; Motyka, P.; Gaebler, M.; Nikulin, V.V.; Villringer, A. Heart–brain interactions shape somatosensory perception and evoked potentials. Proceedings of the National Academy of Sciences 2020, 117, 10575–10584. https://doi.org/10.1073/pnas.1915629117
11. Benedek, M.; Kaernbach, C. A continuous measure of phasic electrodermal activity. Journal of Neuroscience Methods 2010, 190, 80–91. https://doi.org/10.1016/j.jneumeth.2010.04.028
12. Boucsein, W.; Fowles, D.C.; Grimnes, S.; Ben-Shakhar, G.; Roth, W.T.; Dawson, M.E.; Filion, D.L. Publication recommendations for electrodermal measurements. Psychophysiology 2012, 49, 1017–1034. https://doi.org/10.1111/j.1469-8986.2012.01384.x
13. Mathôt, S.; Vilotijević, A. Methods in cognitive pupillometry: Design, preprocessing, and statistical analysis. Behavior Research Methods 2022, 55, 3055–3077. https://doi.org/10.3758/s13428-022-01957-7
14. Lakens, D.; Scheel, A.M.; Isager, P.M. Equivalence Testing for Psychological Research: A Tutorial. Advances in Methods and Practices in Psychological Science 2018, 1, 259–269. https://doi.org/10.1177/2515245918770963
15. Morsella, E.; Gray, J.R.; Krieger, S.C.; Bargh, J.A. The essence of conscious conflict: Subjective effects of sustaining incompatible intentions. Emotion 2009, 9, 717–728. https://doi.org/10.1037/a0017121
16. Yang, E.; Zald, D.H.; Blake, R. Fearful expressions gain preferential access to awareness during continuous flash suppression. Emotion 2007, 7, 882–886. https://doi.org/10.1037/1528-3542.7.4.882
17. Gayet, S.; Paffen, C.L.E.; Belopolsky, A.V.; Theeuwes, J.; Van der Stigchel, S. Visual input signaling threat gains preferential access to awareness in a breaking continuous flash suppression paradigm. Cognition 2016, 149, 77–83. https://doi.org/10.1016/j.cognition.2016.01.009
18. Blankertz, B.; Lemm, S.; Treder, M.; Haufe, S.; Müller, K.-R. Single-trial analysis and classification of ERP components — A tutorial. NeuroImage 2011, 56, 814–825. https://doi.org/10.1016/j.neuroimage.2010.06.048
19. Holm, S. A simple sequentially rejective multiple test procedure. Scandinavian Journal of Statistics 1979, 6, 65–70.
